# Functional Foods and Lifestyle Approaches for Diabetes Prevention and Management

**DOI:** 10.3390/nu9121310

**Published:** 2017-12-01

**Authors:** Ahmad Alkhatib, Catherine Tsang, Ali Tiss, Theeshan Bahorun, Hossein Arefanian, Roula Barake, Abdelkrim Khadir, Jaakko Tuomilehto

**Affiliations:** 1 Dasman Diabetes Institute, P.O. Box 1180, Dasman 15462, Kuwait; ali.tiss@dasmaninstitute.org (A.T.); hossein.arefanian@dasmaninstitute.org (H.A.); roula.barake@dasmaninstitute.org (R.B.); abdelkrim.khadir@dasmaninstitute.org (A.K.); jaakko.tuomilehto@dasmaninstitute.org (J.T.); 2 Faculty of Health and Social Care, Edge Hill University, St. Helens Road, Ormskirk, Lancashire L39 4QP, UK; Tsang@edgehill.ac.uk; 3 ANDI Centre of Excellence for Biomedical and Biomaterials Research, University of Mauritius, MSIRI Building, Réduit 80837, Mauritius; tbahorun@uom.ac.mu; 4 Diabetes Research Group, King Abdulaziz University, P.O. Box 80200, Jeddah 21589, Saudi Arabia

**Keywords:** functional food, Mediterranean diet, physical activity, polyphenols, green tea, yerba mate, bariatric surgery, nutrition counselling, type 2 diabetes mellitus

## Abstract

Functional foods contain biologically active ingredients associated with physiological health benefits for preventing and managing chronic diseases, such as type 2 diabetes mellitus (T2DM). A regular consumption of functional foods may be associated with enhanced anti-oxidant, anti-inflammatory, insulin sensitivity, and anti-cholesterol functions, which are considered integral to prevent and manage T2DM. Components of the Mediterranean diet (MD)—such as fruits, vegetables, oily fish, olive oil, and tree nuts—serve as a model for functional foods based on their natural contents of nutraceuticals, including polyphenols, terpenoids, flavonoids, alkaloids, sterols, pigments, and unsaturated fatty acids. Polyphenols within MD and polyphenol-rich herbs—such as coffee, green tea, black tea, and yerba maté—have shown clinically-meaningful benefits on metabolic and microvascular activities, cholesterol and fasting glucose lowering, and anti-inflammation and anti-oxidation in high-risk and T2DM patients. However, combining exercise with functional food consumption can trigger and augment several metabolic and cardiovascular protective benefits, but it is under-investigated in people with T2DM and bariatric surgery patients. Detecting functional food benefits can now rely on an “omics” biological profiling of individuals’ molecular, genetics, transcriptomics, proteomics, and metabolomics, but is under-investigated in multi-component interventions. A personalized approach for preventing and managing T2DM should consider biological and behavioral models, and embed nutrition education as part of lifestyle diabetes prevention studies. Functional foods may provide additional benefits in such an approach.

## 1. Overview and Background

The prevalence of type 2 diabetes mellitus (T2DM) is increasing at an alarming rate worldwide, causing a significant increase in premature mortality, co-morbidity, and increased healthcare costs [1]. The future predictions are also gloomy given that 1 in 10 people worldwide is expected to have the condition by 2030 [2]. Common determinants include excess body fat, poor diet, physical inactivity, high blood pressure, and family history of diabetes [1]. Appropriate prevention strategies have primarily focused on lifestyle interventions involving physical activity and diet strategies focused on pre-diabetes and high-risk individuals, and conclusively show a significant reduction in T2DM incidence rate from 28% to 58% around the world [3,4,5,6]. Such importance of lifestyle prevention makes it essential to investigate the protective role of healthy nutrients and foods. The term “functional foods” has been coined indicating that such foods have been scientifically proven to have potential health benefits. This review investigates the health protective effectiveness of functional foods, either alone or when combined with physical activity in T2DM prevention. It will also shed some light on how such lifestyle preventative benefits can fit within individualized and localized multi-component prevention models.

All foods with biologically active ingredients are considered functional because of their association with physiological health benefits related to the prevention of several chronic diseases such as T2DM, and a simple online search on PubMed with keywords “diabetes” and “functional food” revealed over 1200 studies on the subject. Although the term “nutraceuticals” often refers to active ingredients found in functional foods, and involves extracting, purifying, concentrating and assaying such ingredients, it is important to differentiate between the terms “functional foods” and “nutraceuticals”.

Mounting clinical evidence demonstrates that T2DM and its associated complications can be prevented or delayed in high risk individuals through regular intake of foods that can be considered functional and impact glycemic control, blood pressure regulation, activation of antioxidant enzymes, gut microbiota, and suppress over production of pro-inflammatory cytokines during diabetes [7]. Additionally, the use of functional foods as a complement therapy for prevention and management of diseases has steadily increased over the past few decades as a means of promoting health and emotional well-being, and has been increasingly applied in cases where patients seek relief of symptoms associated with chronic illness and side effects of conventional medication [8].

A variety of nutrition profiles comprising functional foods have been recommended in healthy meal plans to protect and manage T2DM, such as a Mediterranean Diet (MD), which has been highly rated on the recent American Diabetes Association recommendations for T2DM prevention and management [9], because of the established evidence about MD association with reduced mortality and reduced T2DM incidence [10,11,12]. MD food components may provide a model for their joint effectiveness in preventing T2DM. Some attributed the health protective benefits of MD to the polyphenol content present in MD components such as fruits, vegetables, olive oil, and tree nuts [13,14]. However, traditionally MD or a similar style of diet is considered one of the healthiest diets for human longevity based on epidemiological studies associating health risk-reduction with MD style rather than a single component [10,11,12,15].

Whether the positive functional properties are explained by one or more specific active ingredients, and how effective such properties can be when combined with various physical activity patterns as part of a lifestyle prevention, is under-investigated. Selected physiological responses which can aid T2DM prevention when such foods are either consumed, supplemented, or extracted for their added benefits will be discussed here. The protective role of functional foods in prevention of T2DM will also be discussed as part of a lifestyle intervention which integrates behavioral, biochemical, and physiological models as part of an individualized and localized multi-component model.

## 2. Mediterranean Diet Components as a Model for Functional Foods to Prevent and Manage Diabetes 

MD components consist of high intake of plant-based foods such as fruit, vegetables and legumes, moderate intake of fish and dairy products, and low intake of red meat and red wine [16]. The use of herbs and spices can also be included in place of salt [17]. Collectively, these components are traditionally consumed in regions bordering the Mediterranean region, but adherence is dwindling with the spread of westernized diets in such regions [18,19,20]. Therefore, MD components are not exclusive to any geographical region, and there are promising findings about the implementation of MD in non-Mediterranean regions [21,22].

Functional foods present within the MD containing polyphenols, terpenoids, flavonoids, alkaloids, sterols, pigments, and unsaturated fatty acids play an important role in maintaining wellness, and contribute to preventing cancer, depression, T2DM, obesity, asthma, and cognitive decline [23,24]. Specific to T2DM, reported actions of such foods include an enhanced anti-oxidant, anti-inflammatory and anti-cholesterol properties, enhanced insulin sensitivity and reduced resistance, all considered integral parts of the prevention, management, and treatment of T2DM [25].

Epidemiological studies have long shown an inverse relationship between MD consumption and incidence rate of T2DM [26] and gestational diabetes [27]. Additionally, several recent systematic reviews and randomized controlled trials have demonstrated better T2DM management, and enhanced metabolic state with high-risk individuals, including impaired fasting glucose (IFG), impaired glucose tolerance (IGT) and metabolic syndrome, associated with MD consumption [11,25]. For example, MD compared with control diets has been shown to reduce glycosylated hemoglobin A1c (HbA1c) levels by 0.30–0.47% in T2DM patients [17], and longitudinally is associated with 14.7% and 5% reduced reliance on medication at one and five years post-diagnosis, respectively, compared with a low-fat diet [28]. Prospective analysis of the PERIMED study (Prevención con Dieta Mediterránea) results of one to five years have also shown an inverse T2DM incidence rate associated with MD consumption compared with low fat diet [11,12]. Recent meta-analyses have demonstrated that adherence to MD components of fruit, vegetables, and legumes (measured by MD 1–9 adherence score, and a 136-item food frequency questionnaire) [16] reduces incidence rates irrespectively of obesity changes (indicated by Body Mass Index; BMI) during 9.5-year follow-up, suggesting that MD may attenuate the adverse effects of obesity on the risk of T2DM [29]. Indeed, MD is not a calorically-restricting diet, since some of its central elements are high in energy, especially olive oil and nuts [11,30,31]. Thus, it is important to note that MD is better at lowering T2DM risk irrespective of weight-loss, and that this can be done without necessarily restricting energy intake. 

The benefits of MD components in T2DM have been attributed to specific nutraceuticals within MD food components including monounsaturated fatty acids (MUFA) such as oleic acid in olive oil, omega-3 polyunsaturated fatty acids (e.g., alpha-linolenic acid) found in tree nuts such as walnuts [12], eicosapentaenoic acid (EPA) and docosahexaenoic acid (DHA) found in oily fish, high amounts of flavonoids and antioxidants found in fruits and vegetables [32], and high amounts of fiber found mainly in cereal and whole-grain foods with a low glycemic index (GI) [33,34]. For example, some studies have underlined the importance of olive oil fatty acids—including oleic acid, phytosterols (Beta-sitosterol), antioxidants (alpha-tocopherol)—and plant polyphenols in reducing inflammation and oxidation, and determining improvements in the endothelial micro- and macro-vascular function [13,14]. Such effects are known to have preventative roles in both T2DM and cardiovascular disease (CVD). Others highlighted the importance of fruit and vegetable intake to reduce T2DM risk [32], and conversely low intake of such nutrients is linked with and increased disease risk and even mortality [35].

It is not possible to attribute T2DM risk-reduction benefits to a single functional food or a nutraceutical in MD. Epidemiological studies attempting to link specific MD components to T2DM risk-reduction have found conflicting associations [30]. For example, omega-3 fatty acids, obtained from fish and seafood were only associated with reduced T2DM risk in Asian populations, but not in European or North American populations [30,36]. Others have also shown that longitudinal adherence to low fat diets did not lower T2DM or CVD risk in postmenopausal women [37]. There may be some key functional MD components such as extra-virgin olive oil and tree nuts that have been associated with metabolic mechanistic protective effects such as reducing serum C-reactive protein (CRP), interleukin-6 (IL-6), and endothelial and monocyte adhesion molecules in high-risk men and women [38].

It is plausible to attribute the T2DM protective benefits of MD (improved insulin resistance, glucose control, and other cardiometabolic risk factors) to the polyphenolic content—especially flavan-3-ols—that are present within MD food sources (fruits, vegetables, whole grains, and legumes), and also within drinks and beverages (tea, coffee, red wine, and cocoa) [39]. Clinical evidence has been reported concerning the effectiveness of polyphenol lignans-rich foods (such as flaxseeds) in reducing insulin, glucose, and CRP levels and improving homeostatic model assessment index of insulin resistance (HOMA-IR) in selected patient groups, and supporting epidemiological evidence was also reported for total flavonoid intake association with T2DM risk [39]. Polyphenol-rich olive products—including olive leaves, their crude extract, and extra virgin olive oil—were also reviewed elsewhere for their partial effective role on aspects of the metabolic syndrome [40]. Whereas, non-flavonoid polyphenolic compound hydroxytyrosol, the main polyphenol of olive oil, has been shown to improve the lipid profile, glycaemia, and insulin sensitivity, and counteract oxidative and inflammatory processes [38], and resveratrol (found in grapes, grape products) has been shown to increase intra-cellular transport of glucose and reduce insulin secretion, using various animal and tissue models [41], conferring several benefits for prevention and management in T2DM. Nevertheless, the highest amount of dietary polyphenols across different populations are derived from coffee, and from tea in Asia, and both of them are inversely associated with the risk of T2DM [42]. Therefore, each MD component may have unique characteristics and protective benefits, and we recommend following a holistic approach to implement MD dietary components within diabetes lifestyle prevention.

## 3. Preventive Role of Exercise and Physical Activity in Augmenting Functional Food Effects 

Physical activity is an established primary and secondary prevention of mortality, CVD, and diabetes [43], along with diet forms the bases of lifestyle diabetes prevention [3,4,5,6]. Large lifestyle interventions consisting of combining various forms of structured or unstructured exercise with mainly caloric restriction diets have shown up to 58% reduction of the T2DM incidence rate in high risk individuals, especially those with glucose intolerance from different countries including multiethnic American [3], Finnish [4], Chinese [5], and Indian populations [6]. 

Perhaps the recent interest in healthy functional food components such as MD components and their derived nutraceuticals for T2DM prevention makes it important to highlight the complementing protective role of physical activity, which is also part of the MD pyramid [44]. A recent cross-sectional study has shown that amongst older adults (60–80 years), MD consumers are more likely to have an active lifestyle compared with those who consume a western diet [45]. Regular exercise training combined with adherence to MD intake is likely to trigger or augment additional protective functions such as reduced lipid peroxidation and anti-inflammatory functions, which reflect a better microvascular and macrovascular function in high-risk and older populations [21,44,46].

Several additional cardiometabolic benefits have been reported when MD was combined with additional lifestyle components especially exercise and physical activity, compared with either diet alone or exercise alone [44]. For example, it has been demonstrated that CRP, IL-6, interleukin-18 (IL-18), and tumor necrosis factor-α (TNF-α) have adapted differently when patients combined five weeks of MD adherence with an educational weight loss program, compared with MD alone [47]. In particular, they demonstrated a 26% reduction in CRP concentrations and a 10% reduction in an arbitrary inflammatory score that included CRP, IL-6, IL-18, and TNF-α when the group followed MD only. In comparison, when a weight loss program was combined with MD, two-fold reductions in inflammatory plasma IL-6 (−21%) and IL-18 (−15.6%) were found with no significant impact on plasma CRP concentration [47]. Such differences in single or combined anti-inflammatory effects are known to influence insulin sensitivity of improved endothelial function, although the latter did not disclose whether physical activity was part of their weight loss program. More recent interventions in sedentary older adults and in postmenopausal women, which have combined MD with moderate intensity exercise (determined individually based on ventilatory thresholds and rate of perceived exertion) over a two-month period, demonstrated a greater improvement in endothelial microvascular markers compared with exercise alone [21,46]. Such benefits were largely sustained after one-year follow up of the same cardiometabolic outcomes [48], suggesting that adopting such an approach longitudinally can be effective in T2DM prevention. 

The exercise strategy used as part of a lifestyle intervention plays an integral role in augmenting the cardiometabolic protective benefits of diets, and their derived functional foods. Recently, high intensity interval training (HIIT) has been recommended as a time-efficient strategy for T2DM prevention compared with moderate-intensity exercise [49,50]. HIIT training consists of repeated short bouts of intense exercise (usually above 80% of maximal oxygen uptake or age-predicted heart rate) lasting for about a 1–4 min in duration, followed by approximately equal periods of low-intensity exercise. Evidence is emerging of HIIT effectiveness across different patient cohorts including enhanced postprandial glycemic control (75 g, 2–3 h glucose tolerance), hepatic and improved muscle insulin resistance. For example, a reduction in 24 h glucose levels (7.6 ± 1.0 vs. 6.6 ± 0.7 mmol/L) and a reduced 3-h postprandial glucose, and increased muscle mitochondrial capacity (citrate synthase activity and protein content have been reported in 8 T2DM obese patients following six sessions of HIIT (10 × 60-s cycling bouts at 90% maximal heart rate, interspersed with 60 s rest) over two weeks [51]). Others reported an improved HOMA-Index after each session of a 4 × HIIT sessions in 40-years old T2DM patients [52]. However, research is still needed to demonstrate how to define and implement such a strategy as part of a lifestyle prevention and achieve long-term adherence [53]. Whether and how HIIT combined with functional foods would trigger additional T2DM risk-reduction benefits remain a matter for future research. 

Strength training is often recommended for T2DM prevention and management [2], but limited research has tested the effects of combined strength type training with consuming functional foods such as the MD for T2DM prevention. Such a combination is likely to produce a good MD compliance and long-term adherence, when used in high-risk older adults [54]. Strength training has also been reported to reduce the postmenopausal-related vascular risks, including positive effects on adipose biomarkers of arterial stiffness [55]. These T2DM protective effects in high-risk populations can be further enhanced with adhering to healthy functional foods such as those of the MD components. 

A multi-component approach which encompasses behavioral and physical aspects is likely to be more effective than a single component prevention program. For example, a multi-component intervention in people with metabolic syndrome, which combined hypocaloric MD with 12 weeks of moderate-to-heavy exercise training, was more effective than MD alone in enhancing physical aspects (weight loss, physical fitness, and improvement of metabolic syndrome risk factors) and mental domains of health-related quality of life measures (vitality, general physical health, emotional role, and self-perception of health) [56]. Exercise and consuming functional foods or MD-type diet may have reciprocal functions in terms of promoting health risk-reduction outcomes. Understanding how single or multi MD components could trigger additional exercise benefits associated with T2DM prevention require further research. 

## 4. Protective Role of Polyphenols in T2DM 

Polyphenolic compounds are a diverse and heterogeneous group of secondary plant metabolites commonly classified as phenolic acids, flavonoids, stilbenes, and lignans [57]. They are widespread in the human diet, and their average intake has been estimated to be in the region of 1 g/day [58]. Phenolics are generally thought to be poorly absorbed with plasma concentrations rarely exceeding 1 µM following ingestion of a single phenolic compound [59]. Consequently, only a small number of phenolics are considered bioavailable and therefore of potential therapeutic value. Epidemiological and experimental evidence over the past decade indicated the potential antioxidant defense role in preventing several chronic diseases that are often characterized by an increased production of reactive oxygen species (ROS), including T2DM [60,61,62,63].

Several mechanisms have been proposed to explain polyphenols’ putative anti-diabetic effects. Tea-specific phenolics—particularly green tea, include (+)-catechin and epigallocatechin gallate (ECGC)—suppress oxidative stress, inflammation, and cell death via activation of the nuclear factor erythroid 2-related factor 2 (Nrf2) pathway, leading to the upregulation of antioxidant response element (ARE) gene expression, and enhanced protective enzymes, and free-radical scavengers [64]. Beyond their significant antioxidant capacity, polyphenols present within cocoa, coffee, and yerba maté include phenolic compounds—such as caffeoyl derivatives, procyanidins, and chlorogenic acid—that have all demonstrated ability to influence insulin sensitivity, vascular endothelial function, fat and carbohydrate metabolism, and inflammatory mediators [65,66,67,68,69].

The mechanisms associated with glucocorticoid metabolism, particularly cortisol regulation of glucose homeostasis, have been described in mediating the association between obesity and cardiometabolic risk factors, including hyperinsulinemia and insulin resistance [70]. For example, increased 11β-hydroxysteroid dehydrogenase type 1 (11β-HSD1) activity has been implicated in several metabolic disorders, including T2DM [71]. Specific phenolics, especially ECGC have demonstrated ability as a highly efficacious inhibitor of the cortisol producing enzyme 11β-HSD1 in experimental model systems [72], and consumption of phenolic-rich cocoa and pomegranate regulate cortisol metabolism in clinically obese and overweight populations [73,74]. The potential mechanism of inhibition has been ascribed to their ability to directly bind to the active site of the 11β-HSD1 receptor [72]. These findings demonstrate the potential role of phenolics as novel inhibitors of human 11β-HSD1 and suggest an association between cortisol, glucose, insulin, blood pressure, and lipid profile which may be important in our understanding by which polyphenols influence metabolic parameters in relation to T2DM. 

The anti-diabetic effect of polyphenols, particularly flavonoids within selected functional foods, is promising. Nevertheless, nutritional strategies focusing on modulating T2DM and their comorbidities warrant further investigation, with a particular focus on their bioavailability and bioactivity of metabolites. 

## 5. Clinical Role for Herbal Ingestions in T2DM Prevention and Management 

Effects of numerous herbs and plants have been reviewed for their anti-diabetic functions, including those traditionally used amongst many cultures for centuries. Common ones such as (aloe vera, bilberry extract, bitter melon, cinnamon, fenugreek, ginger, and okra) are already recommended for use on national T2DM prevention guidelines [75]. Nonetheless, recent reviews have listed numerous functional foods and herbs that have been clinically tested and showed various degrees of effectiveness in preventing and managing T2DM. Examples include fukugetin, palmatine, berberine, honokiol, amorfrutins, trigonelline, gymnemic acids, gurmarin, phlorizin, aloe, banaba, bitter melon, caper, cinnamon, cocoa, coffee, fenugreek, garlic, guava, gymnema, nettle, sage, soybean, green and black tea, turmeric, walnut, and yerba maté [76]. Whilst some focused on reviewing the effects of ethanol extracts and crude polysaccharides of complementary medicinal herbs such as Chinese traditional herbs [77]. Reported functions of such ingestions include inhibition of α-glucosidase and α-amylase; effects on glucose uptake and glucose transporters; modification of mechanisms mediated by the peroxisome proliferator-activated receptor; inhibition of protein tyrosine phosphatase 1B activity; modification of gene expression and activities of hormones involved in glucose homeostasis—such as adiponectin, resistin, and incretin; and reduction of oxidative stress [76]. Nonetheless, the mechanistic characteristics of each herb are beyond the scope of this review. The focus is rather on taking a combined lifestyle approach, especially if selected ingredients are combined with exercise, and if an intervention is based on individual characteristics and needs. We therefore highlight selected examples of well-designed trials which evaluated the effectiveness of natural herbs or local foods in preventing and managing T2DM. 

Epidemiological studies have demonstrated various positive associations between herbal tea ingestions and disease prevention including T2DM [64,78]. Typical popular herbal teas including black and green teas native primarily to south Asian countries are now consumed worldwide, and yerba maté, native to South America is now consumed by millions of people in North America, and parts of Europe and the Levant [78,79]. Populations who may be predisposed to diabetes may significantly benefit from the use of herbal tea ingestions, which may be available or produced locally. For example, Mauritian black and green teas—high in polyphenolics—have shown to have potent properties in a Mauritian population, who are predisposed to T2DM [2,80].

In number of randomized and clinical trials, it was shown that CRP levels are reduced by Mauritian tea intake levels in humans [80]. Other reported benefits are fasting blood plasma levels of glucose (−18.4%), triglyceride levels (−35.8%), LDL/HDL plasma cholesterol ratio (−16.6%), with a significant rise in plasma antioxidant propensity (ferric reducing antioxidant power (FRAP): 418%) in a normal healthy population [81]. Three daily cups of green tea were found to reduce waist-hip ratio and fasting plasma glucose in women and suppress mean arterial pressure in men and women after 14 weeks [81]. It also reduced alanine aminotransferase of women by 13.0% while increasing the antioxidant capacity of both men and women by 2.7% and 5.1%, respectively [82].

Complementing clinical trial findings with molecular cellular work contributes to understanding the biological mechanistic insights of tea prophylaxis. The most prominent prophylactic characteristics of Mauritian green tea stem from their antioxidant polyphenolics ranked in the following decreasing order (for total polyphenolic compounds and anti-oxidant capacity): procyanidin B2 > (−)-epigallocatechin gallate > (−)-epigallocatechin > (−)-epicatechin gallate > (−)-epicatechin > (+)-catechin > gallic acid. These were demonstrated to interact with ROS and redox active transition metal ions using a multi-antioxidant assay system [78]. As such, green tea could affect, through its antioxidant and prooxidant activity, the energy metabolism of HEK-293 cells in an oxidative stress-induced diabetic milieu [83]. A recent study comparing the suppressing effects of black and green teas on advanced glycation end products (AGEs) formation and AGEs-induced oxidative stress in 3T3-L1 preadipocytes indicated that both beverages afforded comparable level of protection at cellular level against glycation while black tea exerted highest carbohydrate hydrolyzing enzymes inhibitory activity, thereby confirming an antidiabetic potential [84]. Nonetheless, other AGE reduction mechanisms addressing the pathophysiology of T2DM, remain to be tested in future studies. 

Embedding clinical nutrition findings using herbal ingestions into T2DM lifestyle prevention and management strategies requires consideration of several behavioral lifestyle components, including exercise, energy intake and expenditure, and psychomotor behaviors. In studies using different groups in men and women, positive metabolic, satiety, and mood-state effects have been found following 1–3 h of ingesting 1–2 g of yerba maté [69,85,86]. Yerba maté acute effects included increased fatty acid oxidation (FAO) and energy expenditure from fatty acid oxidation (EEFAO) at various exercise intensities when ingested alone [81] or when yerba maté was combined with a proprietary thermogenic blend of 1.5 g dose containing (green tea extract, yerba maté, guarana seed extract, anhydrous caffeine, saw palmetto, fo-ti, eleuthero root, cayenne pepper, and yohimbine HCI) ingested before moderate exercise [86]. Both studies used mixed gender samples and showed an augmented FAO during low-to-moderate intensity exercise of 24% and 26% in yerba maté compared with placebo respectively. These positive exercise-dependent effects were complemented with several positive effects on mood state (focus, energy, and concentration), and appetite and satiety measures (hunger, prospective eating, and desire to eat) in both resting and exercise conditions (e.g., 23% increase in FAO) following 2 g ingestion of yerba mate compared with placebo in active female participants [69]. Positive behavior and metabolic changes related to nutrient intake and physical activity outcomes are essential for designing an optimized lifestyle prevention for both metabolic health and exercise fat-loss outcomes. 

These studies suggest that the use of popular herbal teas (e.g., green tea, back tea, and yerba maté) have direct and indirect protective outcomes for T2DM. However, we recommend embedding clinical findings into lifestyle intervention studies, involving behavioral components, especially exercise, and to test differently the effectiveness and safety of different doses among high-risk populations.

## 6. The Use of Omics in Detecting the Inter-Individual Functional Food Effects 

There is a need to design individualized and locally-tailored lifestyle and physical activity recommendations to prevent, treat, and manage diabetes where lifestyle is a major risk factor. Such interventions could be empowered using holistic approaches such as “omics”. The term “omics” has been recently defined based on biological profiling of individuals’ molecular characteristics such as genetics (DNA sequence), epigenetics (DNA modification), transcriptomics (gene expression), proteomics (protein products of coding genes), and metabolomics (metabolite products of metabolic pathways), and even microbiomics (bacteria species interacting with host) in multiple types of tissues [87,88]. For example, diabetic patients generally have higher basal energy expenditure and lower activity energy expenditure that is linked to the physiology of the disease and behavioral components, making the investigation of these interactions more difficult [89].

Utilizing the omics approach can help in understanding the effects of functional foods as part of lifestyle prevention of T2DM and associated metabolic disorders. For example, inflammatory mediators (e.g., IL-6, TNF-α, GRP78) and genes expression (e.g., DUSP1) have been linked to differential human individual responses to a lifestyle exercise intervention [90,91]. The latter studies found that the expression levels of IL-6, TNF-α, and DUSP1 were decreased in some but not all obese individuals who followed a 12-week exercise intervention, and similarly only some subjects displayed an improvement in the profile of lipids (LDL, HDL, TG, cholesterol) and glucose (HbA1c and fasting blood glucose), despite no BMI overall change. Thus, subgroups of responders and non-responders to exercise were clustered based on selected omics. Whether such differential effects can be observed following nutritional interventions is under-investigated. So far, only a limited number of studies have applied the omics approach to designing nutritional and lifestyle interventions against diseases such as diabetes [92] or non-alcoholic fatty liver disease (NAFLD) and non-alcoholic steatohepatitis (NASH) [93]. Nutritional studies which utilized omics approaches in relation to how dietary patterns and particular nutrients modulate the risk of T2DM, and focusing in potential specific markers which might differentiate responder from non-responder subjects have been reviewed elsewhere [92]. For instance, Dhtkd1 gene defects, involved in mitochondrial lysine metabolism, were reported to affect the insulin sensitivity and glucose levels in animals [94]. In another animal study, Sptlc3, Klf14, Degs1, Npc and Cbr1 genes were identified to interfere with dietary response and they could be used to predict the interplay between obesity and dietary responses [95]. Since these studies have only used animal models as study targets, human research is needed to integrate the omics determinants to personalize diet, exercise, and a combined lifestyle interventions to prevent and manage T2DM.

## 7. Metabolic Surgery Outcomes and Functional Foods in T2DM Management 

Bariatric surgery (metabolic surgery), commonly used restrictive (e.g., sleeve gastrectomy; SG) and combined (e.g., Roux-en-Y-gastric bypass; RYGB) procedures, is considered one of the most effective treatments for morbidly obese (BMI > 40 kg/m^2^) or obese (BMI = 35–40 kg/m^2^) patients with co-morbidities such as T2DM, hypertension, dyslipidemia, obstructive sleep apnea, obesity hypoventilation, gastroesophageal influx disease, asthma, venous stasis, polycystic ovary syndrome, and pseudotumor cerebri [96]. Some even consider it as a standard option for obese T2DM patients with BMIs as low as 30 kg/m^2^ [97], hence it would be interesting to study metabolic surgery in the context of functional foods. 

Recent clinical data revealed several metabolic benefits in patients with T2DM such as achieving glycemic control, sustained weight loss, and reducing diabetes complications [97,98,99]. The increase in GI tract hormones (such as GLP-1, GIP, PYY, and cholecystokinin) [100,101]; increase in levels of adiponectin [100], elevated lipid oxidation, branched-chain amino acid levels and bile acid production [102,103,104]; and decrease in levels of oxyntomodulin [105], leptin [100], meal-induced ghrelin release [106], circulating free fatty acids [103], Orexin A [100], level of chronic low-grade inflammation [107], activity of digestive vagal afferents, and change in the intestinal microbiome by normalizing obesogenic gut microbiota were observed after bariatric surgery [108,109]. Whether and how such observed metabolic outcomes can be influenced by nutritional intake or specific lifestyle factors is very much under-investigated. 

In terms of eating behavior, it has been reported that bariatric surgery could benefit in improving eating behavior such as binge eating disorder, uncontrolled eating, night eating syndrome, grazing, reduced meal size, increased meal frequency, meal taste and smell, accelerated gastric emptying half-life, decreased T-lag phase duration [110,111,112,113]. However, deficiencies in protein, iron, zinc, copper, calcium, selenium, magnesium, folate, and vitamin B12, B1, D, ascorbic acid, and carotene have been reported in post-surgery cases [114,115,116,117,118]. For example, fat-soluble vitamin deficiencies are reported in malabsorptive procedures and thiamine deficiency was reported in cases with frequent nausea and vomiting [114,119]. Post-operatively reduced food intake, suboptimal dietary quality, altered digestion and absorption, and non-adherence with supplementation regimens are known as potential reasons for nutrient deficiencies after bariatric surgery. Factors related to reduced appetite and increased satiety have also been reported [112,120]. Perhaps nutrition education encompassing dietary functional foods could play important in magnifying the positive eating behavior and overcome nutrients deficiencies observed after bariatric surgery.

The success rate for metabolic surgery in remission or improvement in T2DM (≈78–86%) and in achieving weight loss (≈56%) [121,122] has been explained by age, gender, and genes involved in the metabolic regulation (e.g., single-nucleotide polymorphism, SNPs) [123,124,125,126]. However, it is also documented that 30–50% of patients in whom metabolic surgery was performed failed to achieve their weight-loss goal and 20–25% of cases regained their weight within 10 years [121].

The positive metabolic outcomes associated with metabolic surgery, particularly anti-oxidant and anti-inflammatory benefits, could be further enhanced using herbs and functional foods, especially given their already common use among obesity patients. However, issues related to safety and under-reported use of such foods need to be carefully addressed with such patients [127]. To our knowledge, no research has yet been conducted in this area and further research is warranted. 

## 8. Importance of Education and Counselling in Diabetes Prevention and Management 

It is important to adapt individualized tools to make behavioral changes as part of lifestyle diabetes prevention. Such tools would enhance the outcomes associated with nutritional interventions, including those involving adopting new dietary approaches, such as adherence to non-geographical dietary style [21,22,128]. 

Dietary counselling and education is an integral part in both screening and evaluating the dietary behavior for people with diabetes [129]. It can tease out behaviors related to food and beverage consumption, likes and dislikes, food allergies, and assessing magnitude of reliance on alternative medicine whether in the form of consuming dietary supplementation or natural food products known locally to contribute to glycemic control. It also equips diabetic people with tools and skills needed to better manage their diabetes and prevent development of related co-morbidities and improve their quality of life [130]. A systematic review investigating the effectiveness of dietetic consultations on 5500 adults in primary healthcare settings reported fair evidence (Grade II) in improvement in diet quality, weight loss, and diabetes outcomes [131]. Similar findings were reported by Finnish investigators with a target population of pregnant women with gestational diabetes [132]. Group education have also demonstrated significantly improved scores on quality of life when compared to control groups as shown in adults with type 1 diabetes attending the Dose Adjustment for Normal Eating (DAFNE) program [133] and people with T2DM attending the Diabetes Education and Self-Management for Ongoing and Newly Diagnosed (DESMOND) program [134]. For example, integrating psychological approaches such as using motivational interviewing and cognitive behavior therapy in 19 DAFNE-Hypoglycemia Awareness Restoration Training (DAFNE-HART) participants along with diabetes education led to changes in hypoglycemia awareness [135].

Currently, nutrition education relies heavily on teaching caloric and carbohydrate counting; achieved by identifying sources of carbohydrate in local foods, estimating the portions through using measuring cups and smart food scales, and finally finding out the gram values of carbohydrates consumed through using different techniques such as local and international food composition tables and applications and websites [136]. Healthy meal plans such as the MD can also be individualized and tailored to match diabetics’ needs and requirements based on understanding the behavioral barriers and facilitators to MD, such as food availability in local markets [136,137], and reported affordability and local adaptability in non-Mediterranean cohorts [22,138]. Such a behavioral approach is likely to extend and enhance engagement and adherence in multicomponent interventions [21,22].

In addition to counselling on conventional foods, it is also essential to embed an education to manage the use of alternative and complementary medicine. People with diabetes seem to be 1.6-times more likely to revert to complementary medicine [139], with a high reported consumption rate (22.3–82.3%) [140,141]. However, such consumption is under-reported when counselling and education takes place between healthcare providers and patients, reaching less than 50% in some communities [142]. Thus, it is of utmost importance to integrate the multi-component approach—in clinical, consultation, behavioral, motivational, or educational applications—for maximizing management of diabetes and through conventional and alternative medicine where scientific evidence supports it, and minimizing risk from drug herb interaction or improper usage of supplements [143,144]. Furthermore, understanding local availability, differences in use to treat or manage diseases, and culture and practices, should all be considered in a multi-component behavioral model.

## 9. Conclusions 

This review focused on selected physiological responses which can aid T2DM prevention and management when functional foods are consumed either alone or as part of an intervention. MD food components serve as a model for functional foods and can be effectively adopted as part of an individualized and localized multi-component model, integrating behavioral, biochemical and physiological strategies. The anti-diabetic effect of polyphenols, particularly flavonoids within selected functional foods, is promising. Clinical evidence suggests that popular herbal tea ingestion (e.g., green tea, black tea, and yerba maté) and coffee drinking have direct and indirect protective outcomes for T2DM and associated cardiovascular disease. Each MD component may have unique characteristics and protective benefits, but the reviewed evidence suggests following a holistic approach to implement MD dietary components within diabetes lifestyle prevention. Nevertheless, nutritional strategies focusing on modulating T2DM and their comorbidities warrant further investigation, with a particular focus on bioavailability and bioactivity of their metabolites. Reciprocal health risk-reduction functions are expected when physical activity is combined with consuming functional foods. To understand how exercise enhances or triggers additional protective functional food effects requires further research.

Omics determinants help to individualize functional foods, exercise, or combined lifestyle intervention effects to prevent and manage T2DM. However, human studies are limited and this approach is yet to be utilized with combined exercise and diet lifestyle interventions. Future research is also needed to investigate how the positive metabolic outcomes associated with bariatric surgery, particularly anti-oxidant and anti-inflammatory benefits, could be further enhanced using functional foods such as herbs, especially given their already common use among obese and diabetic patients. 

**Figure 1:**
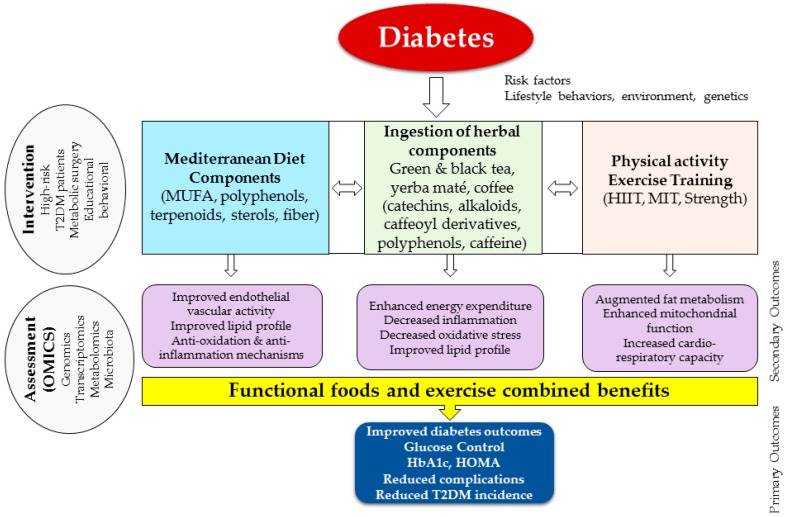
Integration model of functional food in diabetes prevention and management to understand biological processes and improve clinical outcomes. MUFA, monounsaturated fatty acids; HIIT, high intensity interval training; MIT, moderate intensity training; HbA1c, glycosylated hemoglobin A1c; HOMA, homeostatic model assessment; T2DM, type 2 diabetes mellitus.

Integrating a multi-component approach (Figure 1)—clinical, behavioral, and educational—to prevent and manage T2DM through conventional and alternative medicine, requires further scientific evidence to support it and to minimize risk of drug-herb interaction. Furthermore, understanding local availability, differences in use to treat or manage diseases, culture and practices, should all be considered in a multi-component behavioral model.
